# Book Reviews

**Published:** 1912-10

**Authors:** 


### BOOK REVIEWS

---

Trespassers Killed on the Railroads—Who Are They? By *Frank V. Whiting*, General Claims Attorney, N. Y. Central Lines; pamphlet issued by General Safety Agt.

In 1911, 10,396 persons were killed by American railroads. Of these, a trifle over half, 5,284, were traspassers and, of the trespassers, about 80% were killed while walking on or crossing tracks. The vast majority of trespassers killed are men—96.5%

(including boys over 15), the remainder consisting of 3% women, ½% small children. It is commonly supposed that most trespassers killed by railroads are tramps but, in the analysis of a thousand cases, 764 were proved not to be and only 50 positively were proved to be tramps. The contention, with which we heartily concur except when we are in a hurry or get caught in the rain in the country or wish to cross a stream, is that railways should be closed to the public like any other property.

---

THE PRACTICAL MEDICINE SERIES. The Year Book Co., Chicago; 1912. (Price for the series of ten volumes, $10.00) Vol. 3, Eye, Ear, Nose and Throat, 368 pages, illustrated, $1.25.

This volume, like others of the series, is a valuable review of periodic literature, and we need say only that the series is maintaining its high reputation.

---

WHAT TO DO IN CASES OF POISONING, *Wm. Murrell, M. D., F. R. C. P.*, London, published by Paul B. Hoeber, New York. 283 pages, $1.00.

At a glance, this book indicates by its pocket-size, a sensible realization of the needs of the profession. General principles, including medico-legal advice, are given briefly at the front and there follows an alphabetic discussion of individual poisonings, every item showing that the author had in mind the saving of life. Could anything be more practical than the classification of poisons: Patient dead (hydrocyanic, oxalic acids, carbon monoxid and dioxid, etc. ; Patient comatose, collapsed, cyanosed, delirious, tetanized, paralyzed, pupils dilated, contracted, skin dry, moist, rash on the skin, etc.? Under each are given the probable poisons and the snap diagnosis can be verfied from the alphabetic description of individual poisonings.

Poisoning cases are emergent. Lives are lost daily because we have forgotten the teachings of more elaborate and nonportable text books, or because we do not happen to think of the possibilities in a particular case. Every general practitioner ought to carry this book in his pocket.

---

SURGICAL AFTER-TREATMENT. By *L. R. G. Crandon, M. D.*, Assistant in Surgery at Harvard Medical School, and *Albert Ehrenfried, M. D.*, Assistant in Anatomy at Harvard Medical School. Second edition, practically rewritten. Octavo of 831 pages, with 264 original illustrations. Philadelphia and London; W. B. Saunders Company, 1912. Cloth, $6.00 net; Half Morocco, $7.50 net.

The size of this work is, at first sight, a surprise and, at second thought a lesson. Here is a large book covering conditions which, in medical essays, are usually dismissed in a few words; which, in many hospitals are reduced to a brief schedule; for which expert counsel is almost never called; the actual care of which is usually left to pupil nurses and internes. Shock, acetonuria, diet, vaccine therapy, bandaging, a good share of genito-urinary lore, are among the subjects considered. The work is too large to review in detail but its importance cannot be overestimated. Every surgeon ought to read and ponder over it. He will find many hints as to why successful operations are followed by a funeral; how statistics can be run up a few per cent; what is said as to after treatment will often throw light on the management of the pre-operative period. While largely obviating the present crying need of medical consultation in surgical cases, this book will also increase the frequency of such consultations by broadening the surgical horizon. We hope that the next edition will change the word "cæcostomy" to typhlostomy.

**Infant Feeding.** By *Clifford G. Grulee, A. M., M. D.*, Assistant Professor of Pediatrics at Rush Medical College, Attending Pediatrician to Cook County Hospital. Octavo of 295 pages, illustrated. Philadelphia and London; W. B. Saunders Company, 1912. Cloth, $3.00 net.

The author has presented a scientific and, at the same time, practical treatise, well illustrated both by pictures and diagrams. What impresses us especially, is the absence of hobbies. For example, he frankly discusses and acknowledges the value of various proprietary foods and gives sensible directions for home modification of cow's milk but none of his illustrations seem to have been made from cuts turned over by commercial houses.

---

**Collected Papers** by the *Staff of St. Mary's Hospital* (Mayo Clinic) for 1911. Octavo of 603 pages, illustrated. Philadelphia and London; W. B. Saunders Company, 1912. Cloth, $5.50 net.

This is a magnificent collection of monographs, largely devoted to the surgery of the gastro-enteric tract. Dr. Wm. J. Mayo contributes a special paper on the Hospitals and Surgical Clinics of France. There is an interesting memorial of the father of Wm. J. and Charles J. Mayo; Dr. Wm. Worrell Mayo, born in England, a student of Dalton, who died in 1911, aged 92.

---

**The Animal Kingdom** Considered Anatomically, Physically and Philosophically. By *Emanuel Swedenborg.* Part on the Organs of Generation, and the Formation of the Fœtus in the Womb, after which follow chapters on the Breasts and the Periosteum. Translated from the Latin by *Alfred Acton.* 398 pages. 8vo. With 10 anatomical plates. Cloth, $3.00 net. Postage, 20 cents. Philadelphia, Boericke & Tafel. 1912.

This book is complete in itself, "part" referring to the standard collection of the author's works in Latin. The translator and publishers have done well to render this classic available and, while it is, of course, superseded by more modern scientific study, it is worth while to keep in touch with the early students and those who do so will be properly humbled at the extent of knowledge obtained in advance of modern apparatus and collective researches.

---

SEXUAL IMPOTENCE. *Victor G. Vecki, M. D.*, San Francisco. W. B. Saunders. 374 pages, $2.75. 4th edition.

This book discusses very frankly and impartially, a somewhat unsavory subject. While nowhere lewd, it contains many statements quite contrary to religious and conventional ideas, especially in that it usually minimizes the unfavorable results of all sorts of practices commonly thought to be highly injurious. Like almost every other book on the subject, it fails to give normal standards of potency, except by implication from some case reports. But, in general, it is definite in its statements and scientific in its point of view and it impresses us as much more valuable than other works in which the symptomatology and subjective experiences of perverts have been presented at length.

---

X-RAY DIAGNOSIS AND TREATMENT. *W. J. S. Bythell, B. A., M. D.*, and *A. E. Barclay, M. D., M. R. C. S., L. R. C. P.*, of Manchester, Eng. Oxford University Press. 147 pages of text, 118 plates, $5.50.

It should be noted that the plates are full pages, on heavy paper, the page numbering referring merely to printed matter so that the book corresponds in size to one of about 500 pages. More than half of the book is devoted to bones and only about a third to visceral diagnosis. So far as fractures, dislocations and disease of bones are concerned, the work is thorough and of high order. It is excellent for the thorax and abdomen, including the pelvis, but the chapters and plates dealing with this phase of Röntgenology do not enter into minutiae and it may fairly be claimed that various American authors have presented more elaborate studies and obtained clearer negatives. Indeed, a more striking exhibition of gastric, colonic and other visceral pictures could be assembled from the work of Buffalo investigators alone. Local pride, however, should not blind us to the real value of this systematic treatise.

---

PATHOLOGY OF THE EYE. *P. H. Adams, M. A., M. B., D. O., F. R. C. S.* Oxford. Oxford University Press. 194 pages, 43 illustrations, $1.50.

This is just what it purports to be, a concise treatise on pathology, with some allusions to histologic technic, bacteriologic and chemic diagnosis but with no attempt to discuss symptomatology, treatment, etc. It is a most valuable work.

---

THE CARE OF THE SKIN IN HEALTH. *W. Allan Jamieson, M. D., F. R., C. P., Edinburgh.* Oxford University Press. 109 pages, illustrated.

This is a very useful treatise, designed mainly as a guide for laymen and laywomen, the illustrations showing massage apparatus and friction devices, and simple formulae for creams, etc., being given.

---

THE SURGICAL CLINICS OF JOHN B. MURPHY, M. D., Chicago; published bi-monthly by the W. B. Saunders Co., Philadelphia, $8.00 per year (6 issues) Vol. 1, No. 3, June, 1912. 174 pages, illustrated, with skiagrams, etc.

This issue deals mainly with unusual cases of fracture but also with various visceal lesions. The value of these clinics to all persons interested, is very great.

---

CHILDREN—THEIR CARE AND MANAGEMENT. *E. M. Brockbank, M. D., F. R., C. P.*, Manchester, Eng. Oxford University Press, 259 pages, unillustrated, $1.50.

"The object of this book is to offer to newly qualified doctors and to mothers and nurses, some practical advice on the everyday care of children at the nursery age," says the author, and from the dedication to M. E. B., we judge that he has had experience at home. Very wisely, "it contains practically no information about medicines, for this concerns the doctor only." It deals not only with diet, and other matters in the care of the new born, but continues to guide the child's life up to the school age, with sensible advice as to bathing, holidays, recreation, kissing and many other details.

---

MANUAL OF SURGERY, by *Alexis Thomson, F. R. C. S.* and *Alexander Miles, F. R. C. S.*, Edinburgh. Vol 2, Regional Surgery. Henry Frowde and Hodder & Stoughton, Edinburgh, Glasgow and London. 4th edition, revised and enlarged, 924 pages, 274 illustrations.

The sub-title of the volume is liable to lead to an under-estimate of its value; it would perhaps be better to state that it is a very broad and complete presentation of surgical problems classified according to regional anatomy. For example, we find salivary calculus, super-numerary mamma, hernia, gastric, intestinal, genito-urinary and other branches of surgery concisely but well discussed. These are merely selections at random to give some idea of the scope of the work. The volume is small, well executed and convenient and the matter is of the highest order.

---

Vol. 3 on Operative Surgery, 365 pages, 220 illustrations.

This volume includes the operative surgery of the blood vessels, nerves, cranial and spinal, bones, tendons and joints, amputations, skull and face, air passages, chest, abdomen and its viscera, systematically arranged by chapters. Anæsthetics are also considered.

REPORT OF THE COMMISSIONER OF EDUCATION (U. S.), for the year ended June 30, 1911. Government Printing Office.

This is a very interesting compilation of statistics of various kinds. As bearing on the prevalent hard times, it may be noted that more than 10% of youths of high school age and a little over 2.5% of those of college age are students. About ½% more are in training as teachers and nearly another half per cent. are studying for some professional career. These proportions are approximate as there are no definite statistics indicating the number of persons of any single age-year.

13,585 men and 7,882 women received bachelors' degrees in 1911, 2,231 men and 689 women received masters' and doctors' degrees on examination. These figures refer to purely educational courses, not to those in professional courses nor in engineering and various minor professional courses such as music, architecture, library work, etc. This is a good showing if the men and women who have protracted their schooling to the age of 22-30 before getting to work, have minds worth cultivating to this degree and if the country needs so large a number of highly trained intellects. But, to support one person in eight or ten up to the age of 18, one in 35 or 40 up to the age of 22; and about one in 50 up to the age of 25 or 30 is a considerable economic handicap.

---

THE SHAKESPEARE MYTH by *Sir Edwin Durning-Lawrence*, pamphlet of 32 pages, illustrated, published by Gay & Hancock, London.

This contains many interesting reproductions of old prints and a resume of the familiar veiled allusions, by numbers, puns on the name of Bacon in the Shakespearian plays, acrostics, and hidden references in contemporary literature, including Bacon's acknowledged works, which an enthusiastic school of critics have assembled to prove the Baconian authorship. All we have to say on the matter is that there is no analogue in all literature of the production of so much material and of so high grade, both absolutely and relatively to the stage of linguistic and literary development at the time, by a single author, to what are commonly regarded as Shakespeare's writings. It is admitted almost unanimously that these writings were by a single author. Some one accomplished this unprecedented task. If it were not for the actual existence of the matter, unquestionably dating to the time of Shakespeare and Bacon, everyone would declare the production of such a mass of literature impossible. If the Shakespearian literature had been presented to the world anonymously, during Shakespeare's lifetime or at any subsequent period and the literary world had been asked to guess what Englishman of the period composed it, the nearly unanmious vote would have been for Bacon. Shakespeare is known to have been a real personage. Excepting a few ancient authors of rather dubious individuality like Homer, there is none in all history who has left as little of his personality and influence aside from his reputed plays, as Shakespeare. So much for the Baconian claim. On the other hand, to ascribe to Bacon the Shakespearian works as well as all his other activities involves quite as much of a miracle as to suppose that they emanated from the otherwise comparatively unknown and unlikely source of Shakespeare himself. The method of "proof" of the Baconians strikes us as puerile, no better in fact than the method proving anything desired from the Bible by putting together bits separated by centuries of time, without regard to context. Anyhow, Bacon had his chance to claim authorship and, if for hygienic motives, he withheld his claim during his life, he might at least have left a memorandum or confided his secret to a friend. If he chose to create the literary side of Shakespeare, the self-effacing citizen of Avon and modest actor, we shall respect his wishes. Beside, we don't want to establish a precedent for the posthumous killing of Galen, Aesculapius and Hippocrates.

---

Gould & Pyle's Cyclopædia of Practical Medicine and Surgery with Particular Reference to Diagnosis and Treatment. This was reviewed in the September issue but the publisher's name was omitted:—P. Blakiston's Son & Co., of Philadelphia.

